# Evaluation of early innate and adaptive immune responses to the TB vaccine *Mycobacterium bovis* BCG and vaccine candidate BCGΔBCG1419c

**DOI:** 10.1038/s41598-022-14935-y

**Published:** 2022-07-20

**Authors:** Manuja Gunasena, Rajni Kant Shukla, Naiquan Yao, Oscar Rosas Mejia, Michael D. Powell, Kenneth J. Oestreich, Michel de Jesús Aceves-Sánchez, Mario Alberto Flores-Valdez, Namal P. M. Liyanage, Richard T. Robinson

**Affiliations:** 1 Department of Microbial Infection and Immunity, The Ohio State University, Columbus, OH USA; 2 Jilin Agricultural University, Changchun, Jilin China; 3 Biotecnología Médica y Farmacéutica, Centro de Investigación y Asistencia en Tecnología y Diseño del Estado de Jalisco, Av. Normalistas 800, Col. Colinas de la Normal, 44270 Guadalajara, Mexico; 4 Department of Veterinary Biosciences, The Ohio State University, Columbus, OH USA

**Keywords:** Immunology, Microbiology

## Abstract

The vaccine *Mycobacterium bovis* Bacillus Calmette-Guérin (BCG) elicits an immune response that is protective against certain forms of tuberculosis (TB); however, because BCG efficacy is limited it is important to identify alternative TB vaccine candidates. Recently, the BCG deletion mutant and vaccine candidate BCGΔBCG1419c was demonstrated to survive longer in intravenously infected BALB/c mice due to enhanced biofilm formation, and better protected both BALB/c and C57BL/6 mice against TB-induced lung pathology during chronic stages of infection, relative to BCG controls. BCGΔBCG1419c-elicited protection also associated with lower levels of proinflammatory cytokines (i.e. IL6, TNFα) at the site of infection in C57BL/6 mice. Given the distinct immune profiles of BCG- and BCGΔBCG1419c-immunized mice during chronic TB, we set out to determine if there are early immunological events which distinguish these two groups, using multi-dimensional flow cytometric analysis of the lungs and other tissues soon after immunization. Our results demonstrate a number of innate and adaptive response differences between BCG- and BCGΔBCG1419c-immunized mice which are consistent with the latter being longer lasting and potentially less inflammatory, including lower frequencies of exhausted CD4^+^ T helper (T_H_) cells and higher frequencies of IL10-producing T cells, respectively. These studies suggest the use of BCGΔBCG1419c may be advantageous as an alternative TB vaccine candidate.

## Introduction

Tuberculosis (TB) is a leading cause of death from an infectious disease^1^. TB is caused by aerogenic transmission of *Mycobacterium tuberculosis* (Mtb), a bacterial species which infects alveolar macrophages and can disseminate to extrapulmonary tissues via lymphatic or hematogenous spread^2^. TB has a spectrum of clinical manifestations that vary depending on the host response, including severe, active, chronic, subclinical and latent forms^3^. Improved socioeconomic conditions, public health practices and the use of effective drug treatment have reduced global TB rates; however, these rates fell short of the World Health Organization (WHO) goal of reversing TB incidence by 2015^4^, and are not on target to achieve WHO benchmarks for ending the TB epidemic by 2030^5^. For these reasons, it is important for there to be a safe vaccine which effectively promotes TB resistance.

The TB vaccine Bacillus Calmette-Guerin (BCG) is a live-attenuated strain of *Mycobacterium bovis* whose human virulence has been attenuated by multiple passages in artificial culture. BCG is administered throughout the world as a neonatal vaccine against severe forms of childhood TB^6^; however, the use of BCG in children is controversial due to its variable efficacy^7^, as well as the number of adverse events which stem from its early immunogenicity^8^. Within the first 2 weeks post-immunization, nearly all BCG recipients experience mild adverse events, ranging from papule formation at the site of vaccination, which may become ulcerated or leave a scar, to swelling of the draining lymph nodes. Severe adverse events include more pronounced forms of skin lesions (e.g. BCG lupus vulgaris), lymphadenitis with suppuration, and osteitis. Due to the number of adverse inflammatory events which accompany BCG-immunization, and increased vaccine hesitancy among parents in TB-endemic countries^9–12^ who are reluctant to expose their children to inflammatory vaccines (regardless of their efficacy), it is important to identify TB vaccine candidates which—relative to BCG—have reduced early reactogenicity but comparable or better protective efficacy against TB.

Recently, the vaccine candidate BCGΔBCG1419c was demonstrated to confer equal and/or better protection against experimental TB compared to its parental strain, BCG Pasteur, in a variety of mouse models (BALB/c^13,14^, C57BL/6^15,16^, and B6D2F1 [C57BL/6J × DBA/2J]^13^), including a type 2 diabetic BALB/c model^17^, while in guinea pigs it was more effective than BCG in reducing hematogenous spread at 2 months post-infection^18^. The *M. bovis BCG1419c* gene encodes a phosphodiesterase activity that hydrolyzes bis-(3′-5′)-cyclic dimeric GMP (c-di-GMP), a molecule which reduces bacterial motility and increases biofilm formation (biofilms are sessile bacteria encased within a matrix of an extracellular polymeric substance)^19^. As a consequence of *BCG1419c* deletion, BCGΔBCG1419c strain has an increased capacity for biofilm production and persists longer in immunocompetent mice relative to BCG Pasteur after intravenous infection^20^. Importantly, BCGΔBCG1419c-immunized mice also exhibit less lung pathology post Mtb-infection, relative to the BCG-immunized mice that were later infected with Mtb^15^. Interestingly, at 60 days post-vaccination, BCGΔBCG1419c-immunized mice showed increased numbers of T CD3^+^ CD4^+^ and of T CD3^+^CD4^+^CD44^+^ cells in BALF obtained from C57BL/6 as compared with parental BCG^16^. Also, in spleens of BALB/c mice, BCGΔBCG1419c increased T CD3^+^CD4^+^IFNγ^+^ and T CD3^+^CD8^+^IFNγ^+^ cells with respect to parental BCG^13^. Moreover, we have observed that BCGΔBCG1419c promote a differential presence in lungs of IFNγ-producing T cells, and activated macrophages in a BALB/c model of active and chronic TB^13^ while it changed the relative presence in lungs of B, CD8^+^, and dendritic cells, in a BALB/c model of TB-type 2 diabetes^17^. All these findings show that BCG and BCGΔBCG1419c affect the relative composition of adaptive immune cells pre- and post-infection in diverse organs and tissues of vaccinated hosts.

Given that BCGΔBCG1419c is an effective vaccine in multiple models of experimental TB, outperforming BCG in preventing TB disease measured as lung pathology, and spread to spleen, we carried out a series of experiments to determine if early immunogenicity of BCGΔBCG1419c is comparable or distinct from that elicited by BCG, the adverse inflammatory effects of which are typically observed within 2–3 weeks post-immunization. Specifically, we immunized wild type mice and a transgenic IL10 reporter mouse strain with BCG or BCGΔBCG1419c, and subsequently performed deep immunophenotyping to comprehensively identify the innate and adaptive immune events that occur within the first 2–3 weeks post-immunization, as well the cellular sources of IL10^21–24^. Our data demonstrate heretofore unknown, early immunological events induced by BCG, as well as phenotypic and functional differences between the immune responses elicited by BCG and BCGΔBCG1419c. The relevance of our data to BCG mechanisms of early immunogenicity, as well as understanding of BCGΔBCG1419c efficacy, are discussed.

## Methods

### Ethics statement

This study and its associated experiments were approved by The Ohio State University (OSU) Institutional Biosafety Committee (IBC), as well as the OSU Institutional Animal Care and Use Committee (IACUC). The study was performed in accordance with relevant institutional guidelines and is reported in accordance with ARRIVE guidelines (https://arriveguidelines.org).

### BCG culture

Frozen vials of *M. bovis* BCG Pasteur strain (BCG) and its isogenic derivative, BCGΔBCG1419c, which lacks a cyclic di-GMP phosphodiesterase encoded by the gene *BCG1419c*^20^, were received from Colorado State University (Ft. Collins, CO) from a previous study^15^, and stored at − 80 °C. Upon thawing, the bacterial suspension (1 mL) was transferred into 9 mL 7H9 media (supplemented with OADC, 0.1% Tween 80), in a sterile 150 × 25 mm screw-capped glass culture tube; a very small, plastic coated magnetic stir bar was added for culture aeration and agitation. This bacteria/7H9 suspension was placed on a magnetic stirrer and cultured for 15 days @ 37 °C 5% CO_2_ (the minimum magnetic force needed to agitate the stir bar was applied during this period). After this initial 15 day period, 5 mL of the bacteria/7H9 suspension was transferred into 50 mL of synthetic Proskauer Beck media (modified from Youmans and Karlson^25^, PB: 0.5% KH_2_PO_4_, 0.5% l-asparagine monohydrate, 0.06% MgSO_4_·7H_2_O, 0.25% magnesium citrate, 2% glycerol, 0.05% Tween 80) in a sterile glass culture bottle and cultured for an additional 17 days @ 37 °C 5% CO_2_ (cultures were gently rocked at 30 RPM during this period). At the end of this 17-day period, BCG and BCGΔBCG1419c were aliquoted and rapidly frozen in screw-capped microcentrifuge tubes at − 80 °C. After 5 days of being frozen at − 80 °C, individual aliquots of BCG and BCGΔBCG1419c were thawed and serial dilutions plated on 7H10 (supplemented with OADC, 0.1% l-asparagine) and TCA (to test for contamination). Colony counts demonstrated both BCG and BCGΔBCG1419c aliquots to be at 5 × 10^4^/mL; TCA plates showed no growth (i.e. neither BCG nor BCGΔBCG1419c contained contaminants).

### Mouse strains

C57BL/6 mice were acquired from The Jackson Laboratory (Bar Harbor, ME); IL10 reporter mice (C57BL/6 background) were generously provided by Dr. Weiguo Cui (Blood Research Institute, Blood Center of Wisconsin, Milwaukee WI)^26^. All mice were maintained within The Ohio State University (OSU) University Laboratory Animal Resources (ULAR), and supplied with sterile and water ad libitum. All methods and procedures were performed in accordance with OSU Institution Animal Care and Use Committee (IACUC) approved protocols and procedures.

### Immunization and tissue collection

On the day of immunization (day 0), aliquots of BCG and BCGΔBCG1419c were thawed and directly loaded into tuberculin syringes. Mice were immunized subcutaneously (s.c.) at the back of their neck with 1 × 10^4^ CFU of either BCG or BCGΔBCG1419c (i.e. 200 µL of a 5 × 10^4^ CFU/mL aliquot). On post-immunization day 14, the cervical lymph nodes and spleen were collected from euthanized IL10 reporter mice and used for flow cytometric analysis. On post-immunization day 18, blood, spleen and lungs were collected from euthanized C57BL/6 mice and used for flow cytometric analysis. As described in our Results, our analysis of IL10 reporter mice was performed before that of C57BL/6 mice (day 14 vs day 18) to determine whether IL10 expression differences precede the phenotypic differences between BCG- and BCGΔBCG1419c-immunized C57BL/6 mice.

### Cell isolation

Spleen and lymph node tissues were gently pressed through a mesh screen using the plunger portion of a 1 mL syringe. Lungs were similarly processed following collagenase/DNase treatment. The resulting cell suspensions were transferred to a 15 mL tube by filtering through 100-micron filter using ice-cold R10 media (RPMI 1640 by Gibco^®^ Thermo Fisher, 10% FBS, 2 mM l-glutamine, 100 U/mL penicillin G, 100 μg/mL streptomycin). Tubes were spun down at 250×*g* for 7 min at 4 °C. The cell pellet was resuspended with R10 media. After discarding supernatant, the cell pellet was washed again and incubated with 1 mL of RBC lysis buffer at room temperature. The RBC lysis reaction was stopped after 10 min using 1 mL of R10 media and spun down at 250×*g* for 7 min at 4 °C.

### Flow cytometry cell staining

Freshly isolated cells from spleen, lung and blood tissues were transferred to pre-warmed R10 media and washed. Cells were then resuspended at 1–2 million cells per ml in R10 media and stimulated in FACs tubes with PMA/Ionomycin (final concentration of 2 μg/mL) in the presence of GolgiPlug, at a final concentration of 10 μg/mL; (BD Biosciences, San Jose, California) for 12 h in a 37 °C and 5% CO_2_ incubator. At the end of the incubation, intracellular staining procedure was performed. The following monoclonal antibodies were used: FITC anti-CD3 (clone 17A2), Alexa Fluor 532 anti-CD4 (clone RM4-5), PE anti-CD127 (clone SB/199), PE-eFluor 610 anti-PD1 (clone J43), PE-Cy5 anti-CD11b (clone M1/70), PerCP-Cy5.5 anti-CD44 (clone IM7), PerCP-eFluor 710 anti-CXCR5 (clone SPRCL5), PE-Cy7 anti-CD117 (clone 2B8), Alexa Fluor 647 anti-CRTH2 (clone No3m1scz), Alexa Fluor 700 anti-TNFα (clone MP6-XT22), Brilliant Violet 421 anti-CD45 (clone 30-F11), Super Bright 436 anti-CD62L (clone MEL-14), eFluor 450 anti-Granzyme b (clone NGZB), BD Horizon BV480 anti-IFNγ (clone XMG1.2), Brilliant Violet 570 anti-CD8 (clone 53-6.7), Brilliant Violet 605 anti-IL17 (clone TC11-18H10.1), Brilliant Violet 650 anti-Gr1 (clone RB6-8C5), Brilliant Violet 711 anti-CD19 (clone 6D5), Brilliant Violet 750 anti-CD27 (clone LG.3A10), Brilliant Violet 785 anti-NK1.1 (clone PK136). Samples were stained with Fixability Viability Dye (Aqua Live/Dead stain) and incubated for 15 min. Then cells were washed and followed by adding of surface antibody cocktail. After incubating for 30 min cells were washed and fixed with fixation buffer (Cat No:420801 Biolegend) for 20 min and permeabilized with 1 × Intracellular Staining Permeabilization Wash Buffer (Cat No: 421002 Biolegend). Recommended amounts of directly conjugated primary antibodies for detection of intracellular antigens were added and incubated for 25 min at 4 °C in dark. All washing steps were carried out at 700*g* for 6 min at 4 °C. All antibodies were previously titrated to determine the optimal concentration. Finally, after washing and filtering cells through strainer capped FACs tubes, samples were acquired on a Cytek Aurora or FACS Canto flow cytometer and analyzed using FlowJo version 10.6.1 (Becton Dickinson, Ashland, OR).

### Statistics

Figures were prepared using GraphPad Prism, version 7. Statistical analyses used the bundled software. Bars in the figures show means plus standard deviations (SD). Numbers shown between data points represent *p* values for the comparisons indicated on the figures. Prior to performing statistical comparisons, the Shapiro Wilk test was used to determine if data distribution was normal. Statistical comparisons involving more than two experimental groups used analysis of variance (ANOVA). All other statistical comparisons used Student's t test. The data shown in each figure are representative of 3 separate experiments, with at least 3 mice per experimental group (PBS, BCG, BCGΔBCG1419c).

## Results

### BCG and BCGΔBCG1419c elicit distinct immune responses early post-immunization.

**Figure 1:**
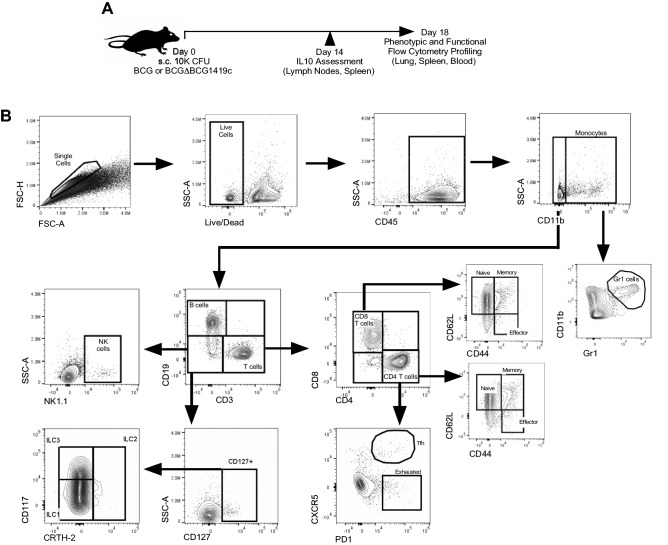
Overview of experiment. (**A**) Vaccination schedule and (**B**) gating strategy to identify innate and adaptive subsets.

To identify the early innate and adaptive immune populations which respond to BCG, as well as identify what effect the deletion of *BCG1419c* has on these responses, we immunized C57BL/6 mice with 10^4^ CFU of live BCG Pasteur and its isogenic derivative, BCGΔBCG1419c (Fig. 1A). Mice immunized with PBS alone served as negative controls. At post-immunization day 18, multiple tissues (blood, spleen and lung) were collected, processed into single cell suspensions and analyzed by multi-dimensional flow cytometry to identify phenotypic and functional differences between innate and adaptive immune subsets (Fig. 1B).

**Figure 2:**
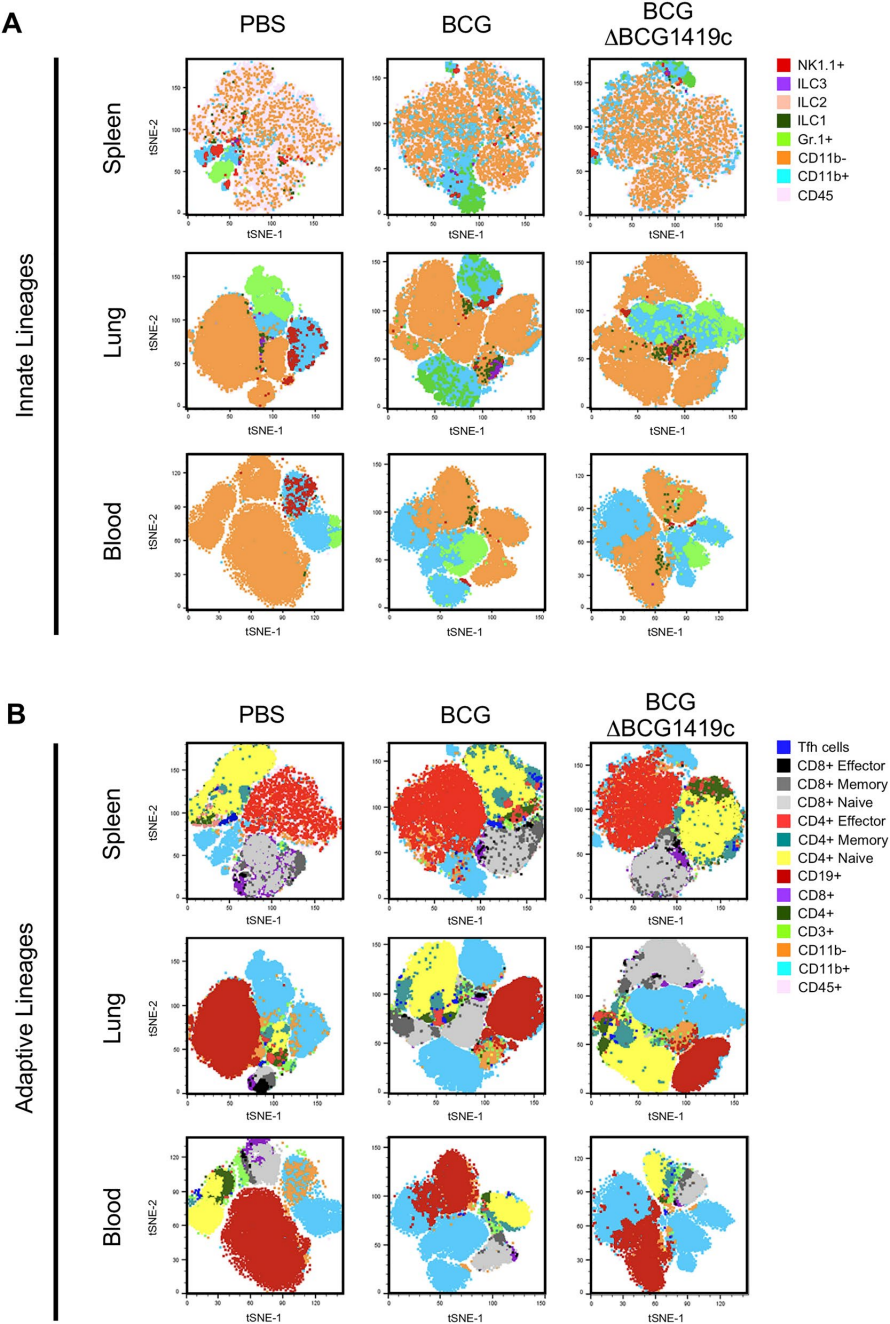
Graph-based clustering of PBS-, BCG- and BCGΔBCG1419c-induced immune populations visualized by T-distributed Stochastic Neighbor Embedding (tSNE). Representative tSNE maps of CD45^+^ cells in the (**A**) blood, (**B**) spleen and (**C**) lungs of control, BCG- and BCGΔBCG1419c-immunized mice. In each t-SNE plot, clusters are color coded and assigned a lineage identification based on the markers for which are defined in Fig. 1B.

To comprehensively assess whether deletion of *BCG1419c* alters the BCG-induced immune landscape in mice, a 27-color flow cytometry panel was designed to characterize the pulmonary and systemic immune populations. We applied conventional flow cytometry and t-distributed stochastic embedding (tSNE) dimensionality reduction plots of CD45^+^ cells from the spleen, lung and blood, which allowed the identification of 19 immune cell populations (Fig. 2). The clustering algorithm allowed for the automatic definition of 8 innate immune metaclusters wherein CD3 and CD19 expression was absent (Fig. 2A), and 11 adaptive immune metaclusters wherein CD3 and CD19 expression was present (Fig. 2B; the same tSNE plots shown in Fig. 2A but with the T- and B-cell overlay). Distinctions between lung, spleen and blood cluster distributions can be observed by comparing the top, middle and bottom rows of Fig. 2A,B, as well as between PBS control, BCG and BCGΔBCG1419c immunized mice (compare left, middle and right columns of Fig. 2). With regards to innate lineages, we observed reductions in NK cells’ representation among CD45^+^ cells in the spleens of BCG and BCGΔBCG1419c immunized mice compared to PBS controls (Fig. 2A upper panel, red data points), as well as the lungs (Fig. 2A middle panel) and blood (Fig. 2A lower panel). Conversely, the representation of monocytes among CD45^+^ was increased in the blood following BCG and BCGΔBCG1419c immunization compared to PBS controls (Fig. 2A lower panel, light blue data points). With regards to adaptive lineages, tSNE analysis further revealed an elevation of B cell cluster in the spleen, as well as elevated CD4^+^ and CD8^+^ T cell clusters in the lungs of BCG or BCGΔBCG1419c immunized group (Fig. 2B).

### BCG and BCGΔBCG1419c elicited innate immune responses

**Figure 3:**
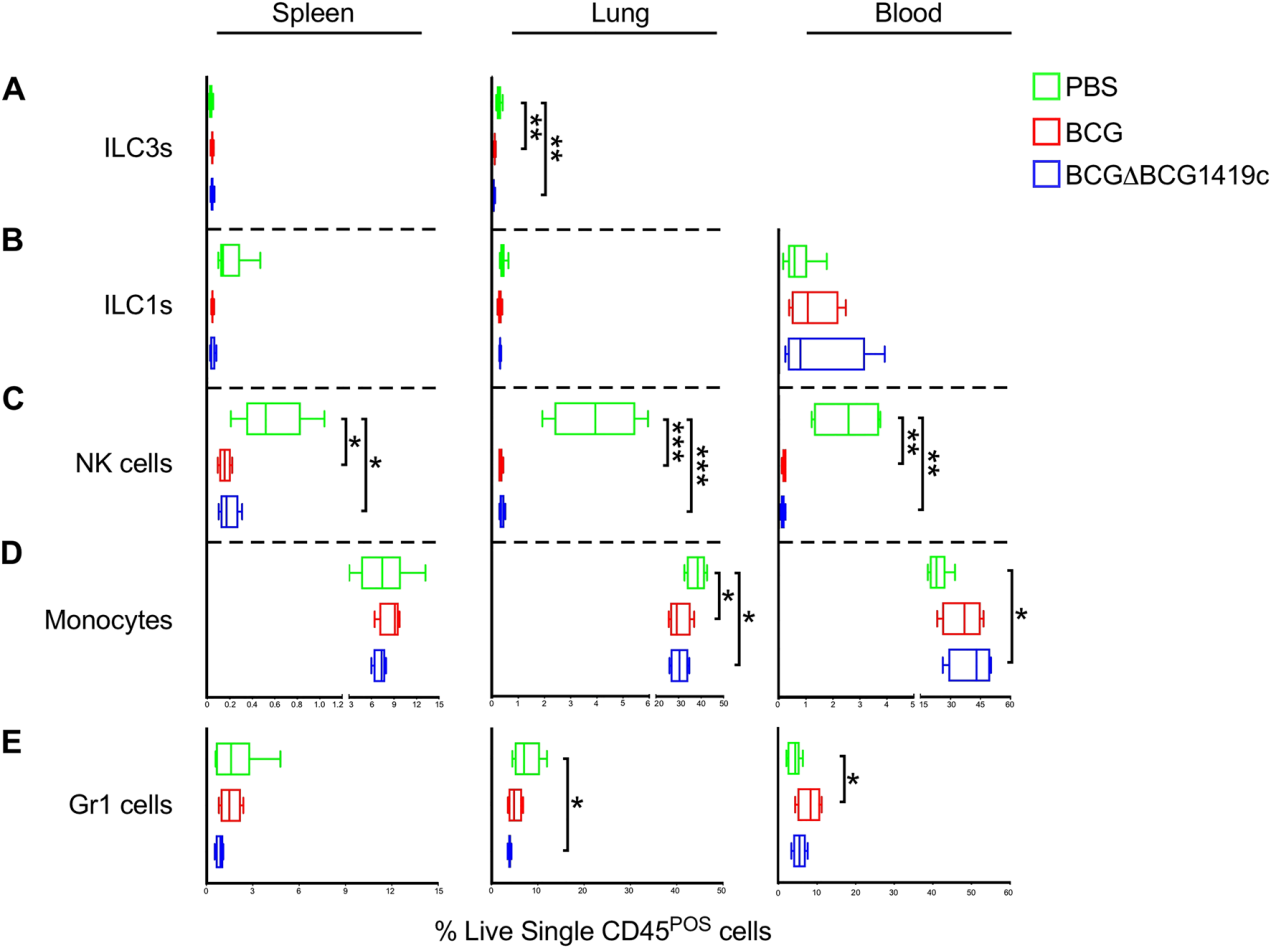
Innate immune subset frequencies in BCG- and BCGΔBCG1419c-immunized mice. Innate immune subset (i.e. ILC, NK cell, monocyte and Gr1 cell) frequencies in BCG- and BCGΔBCG1419c-immunized mice, as well as PBS controls, were measured in the spleen, lung and blood on post-immunization day 18. Shown are the frequencies of (**A**) ILC3s, (**B**) ILC1s, (**C**) NK cells, (**D**) monocytes and (**E**) Gr1 cells in the spleen (left column), lung (middle column) and blood (right column), as gated off of live single CD45^+^ cells. ILCs were gated off CD3^−^CD19^−^CD127^+^, and different subsets were determined according to the expression of CRTH2 vs cKit: ILC1, CRTH2^−^cKit^−^; ILC2, CRTH2^+^cKit^+/−^; ILC3, CRTH^-^cKit^+^. Boxes represent data from 3 mice per group; asterisks represent a statistically significant difference between indicated rows (*p ≤ 0.05; **p ≤ 0.005; ***p ≤ 0.0005).

**Figure 4:**
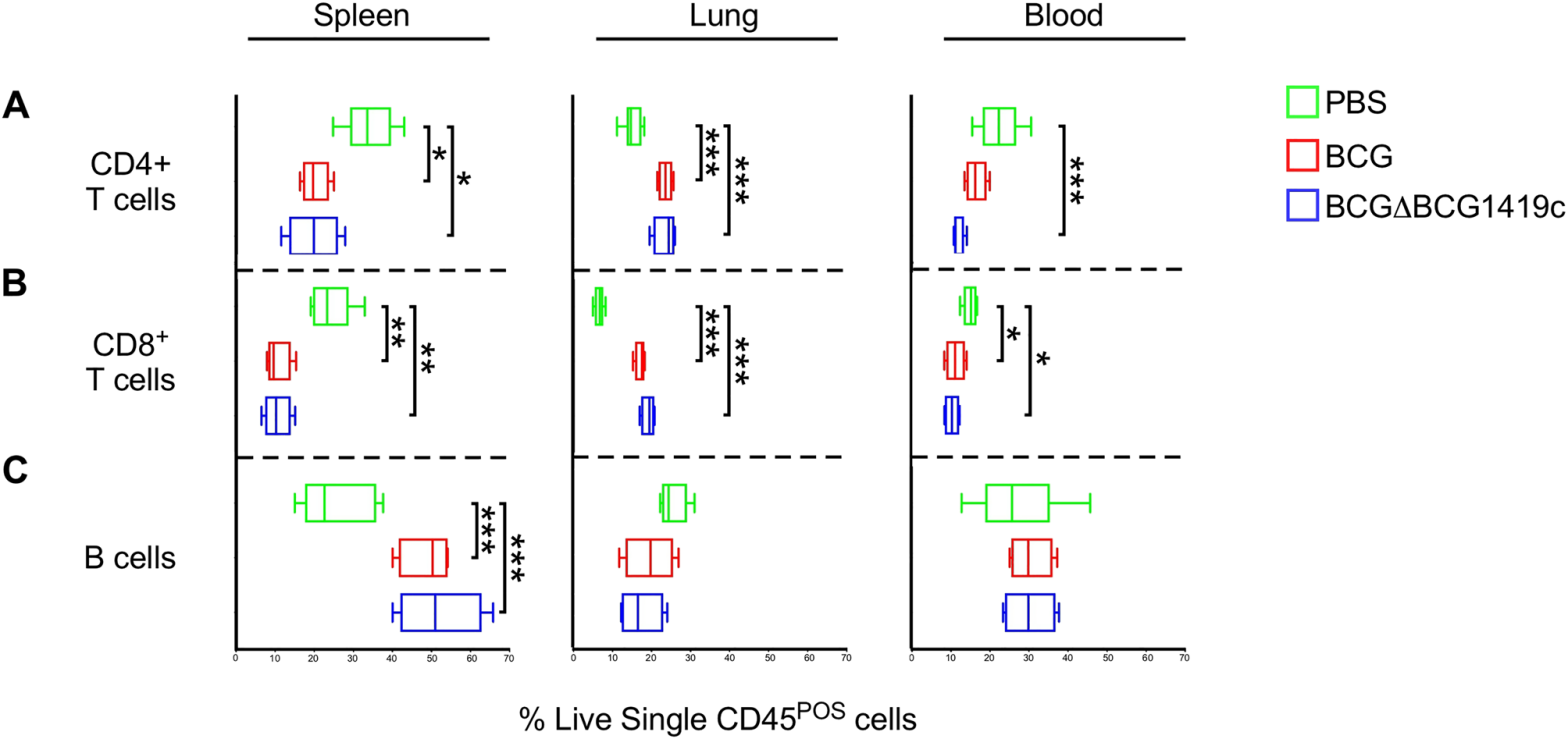
Adaptive immune subset frequencies in BCG- and BCGΔBCG1419c-immunized mice. Adaptive immune subset (i.e. T and B cell) frequencies in BCG- and BCGΔBCG1419c-immunized mice, as well as PBS controls, were measured in the spleen, lung and blood on post-immunization day 18. Shown are the frequencies of (**A**) CD4^+^ T cells, (**B**) CD8^+^ T cells and (**C**) B cells in the spleen (left column), lung (middle column) and blood (right column), as gated off of live single CD45^+^ cells. Boxes represent data from 3 mice per group; asterisks represent a statistically significant difference between indicated rows (*p ≤ 0.05; **p ≤ 0.005; ***p ≤ 0.0005).

**Figure 5:**
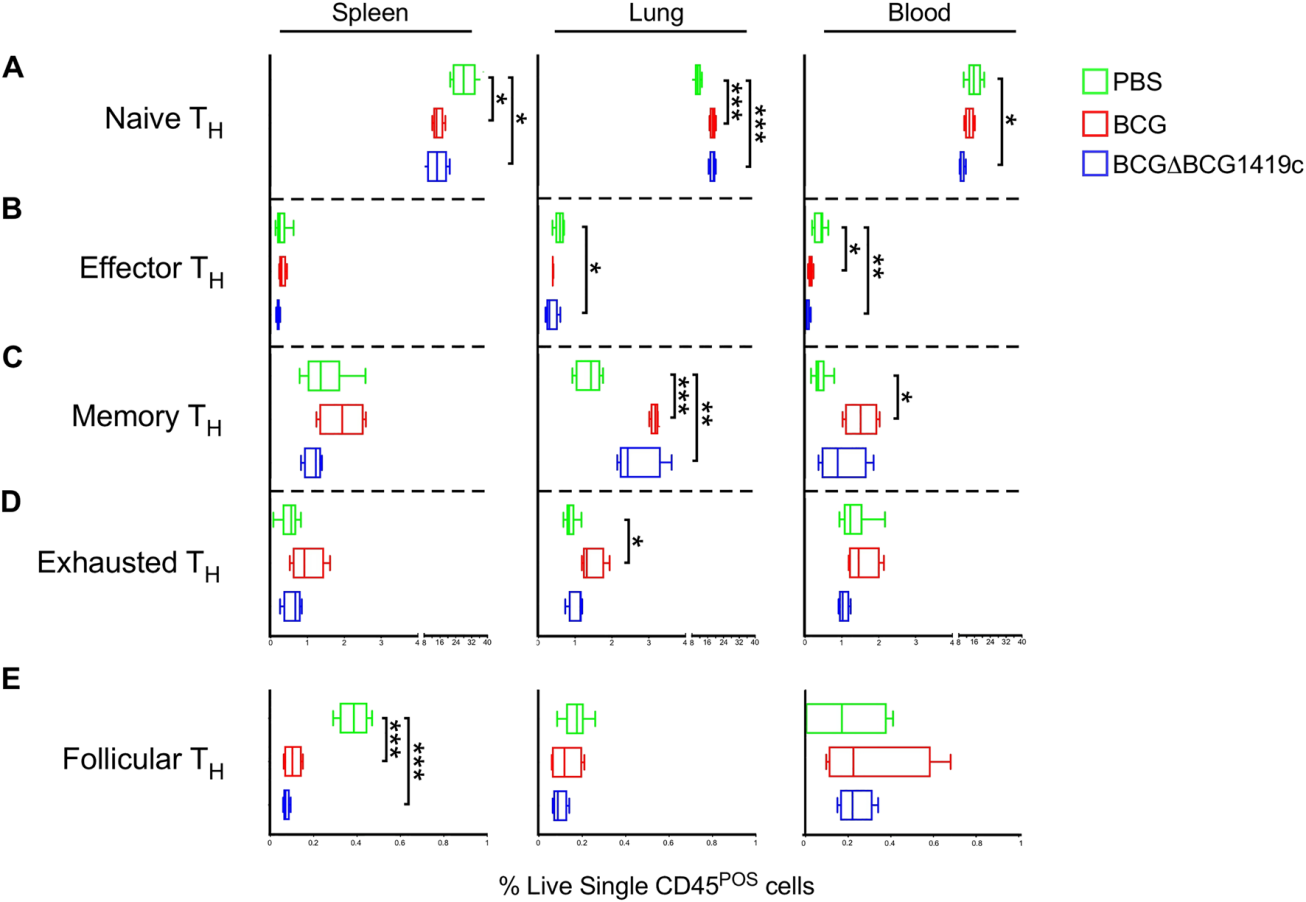
CD4^+^ T cell subset frequencies in BCG- and BCGΔBCG1419c-immunized mice. CD4 T cell (T_H_) subset frequencies in BCG- and BCGΔBCG1419c-immunized mice, as well as PBS controls, were measured in the spleen, lung and blood, based on their expression of CD62L, CD44, PD1 and CXCR5 (naïve, CD62L^HI^CD44^LO^; effector, CD62L^LO^CD44^HI^; memory, CD62L^HI^CD44^HI^; exhausted, PD1^+^; follicular, CXCR5^+^PD1^+^). Shown are the frequencies of (**A**) naïve T_H_ cells, (**B**) effector T_H_ cells, (**C**) memory T_H_ cells, (**D**) exhausted T_H_ cells, and (**E**) follicular T_H_ cells in the spleen (left column), lung (middle column) and blood (right column), as gated off of live single CD45^+^ cells. Boxes represent data from 3 mice per group; asterisks represent a statistically significant difference between indicated rows (*p ≤ 0.05; **p ≤ 0.005; ***p ≤ 0.0005).

**Figure 6:**
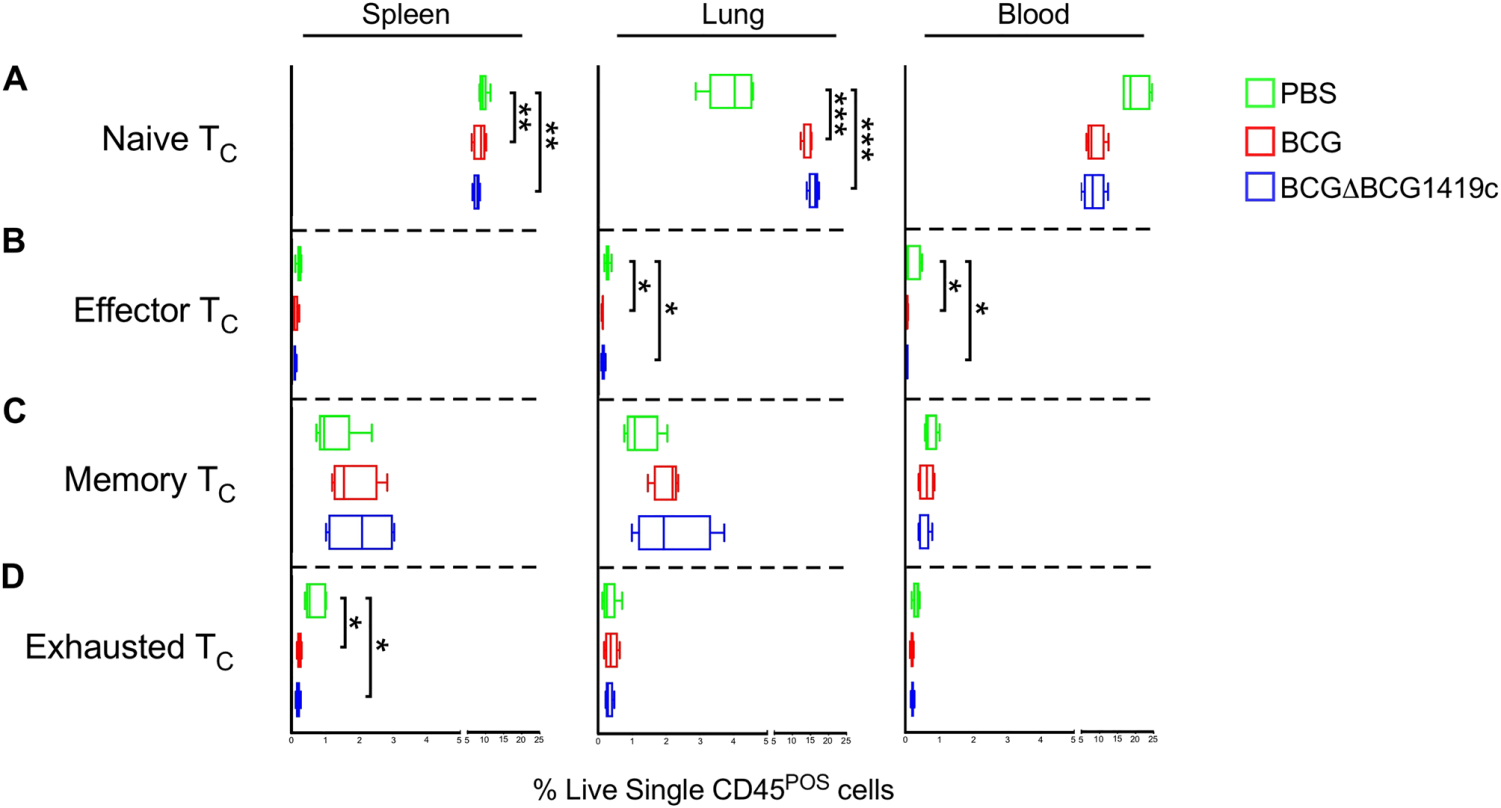
CD8^+^ T cell subset frequencies in BCG- and BCGΔBCG1419c-immunized mice. CD8 T cell (T_C_) subset frequencies in BCG- and BCGΔBCG1419c-immunized mice, as well as PBS controls, were measured in the spleen, lung and blood, based on their expression of CD62L and CD44 (naïve, CD62L^HI^CD44^LO^; effector, CD62L^LO^CD44^HI^; memory, CD62L^HI^CD44^HI^; exhausted, PD1^+^). Shown are the frequencies of (**A**) naïve T_C_ cells, (**B**) effector T_C_ cells, (**C**) memory T_C_ cells and (**D**) exhausted T_C_ cells in the spleen (left column), lung (middle column) and blood (right column), as gated off of live single CD45^+^ cells. Boxes represent data from 3 mice per group; asterisks represent a statistically significant difference between indicated rows (*p ≤ 0.05; **p ≤ 0.005; ***p ≤ 0.0005).

Whereas tSNE analysis permits high-level data visualization, we used traditional flow cytometry analysis to more closely and quantitatively examine the innate and adaptive populations that were differentially affected by BCG and BCGΔBCG1419c. Since tSNE analysis demonstrated the pulmonary and systemic responses to vaccination were distinct, differences among spleen (left panels of Figs. 3, 4, 5, 6), lung (center panels of Figs. 3, 4, 5, 6) and blood (right panels of Figs. 3, 4, 5, 6) were considered separately.

Among the innate lineages (ILC3, ILC1, NK cells, monocytes, and Gr1 cells), nearly all changed in response to BCG and/or BCGΔBCG1419c immunization, the degree to which varied between spleen, lung and blood (Fig. 3). In all tissues, NK cell frequencies declined following BCG or BCGΔ1419c immunization (Fig. 3C), while ILC1 frequencies were unchanged (Fig. 3B). Other changes were tissue-specific: whereas the frequencies of spleen ILC3s, monocytes, and Gr1 cells were unaffected by immunization, their frequencies were decreased in the lung of BCG or BCGΔBCG1419c immunized animals (Figs. 3A,D,E). The only innate frequencies which were positively affected by immunization were blood monocytes in BCGΔBCG1419c immunized animals (Fig. 3D), and blood Gr1 cells in BCG immunized animals (Fig. 3E). Collectively, these data demonstrate that BCG and BCGΔBCG1419c immunization each have generally negative effects on innate cell frequency in the spleen and lung, but unique effects on monocyte and Gr1 cell frequencies in the blood.

### BCG and BCGΔBCG1419c elicited adaptive immune responses

Conventional αβ T cells are critical for the long term efficacy of BCG vaccination^27,28^, and although B cells have historically been considered dispensable for BCG efficacy their presence may shape the T cell response in an antibody-independent manner^29,30^. Lung CD4^+^ and CD8^+^ T cell frequencies increased in BCG and BCGΔBCG1419c immunized animals, with corresponding declines in the spleen and blood (likely a consequence of T cell redistribution from the circulation) (Fig. 4A,B); the ability of subcutaneously administered BCG to increase lung T cell numbers have been previously reported^31^. B cells’ response to immunization was likewise tissue-specific: while no differences were observed in the lung or blood across groups, increased B cell frequencies being observed in the spleens of BCG and BCGΔBCG1419c -immunized compared to PBS controls (Fig. 4C). Collectively, these data demonstrate that BCG and BCGΔBCG1419c immunization causes similar increases in lung T cell and spleen B cell frequencies.

### BCG and BCGΔBCG1419c elicited T cell memory

An important component of BCG-mediated immunity is T cells’ capacity for memory development. Unlike other pulmonary infection models, BCG-mediated immunity lay within T cells that maintain a naïve, antigen-inexperienced surface phenotype^31^. Shown in Figs. 5, 6, respectively, are the frequencies of CD4 and CD8 T cells which exhibit a naïve (CD62L^HI^CD44^LO^), effector (CD62L^LO^CD44^HI^) and central memory (CD62L^HI^CD44^HI^) surface phenotype. The frequency of follicular T_H_ cells (i.e. Tfh cells) was also examined.

Relative to PBS controls, higher proportions of lung CD4 T cells in BCG and BCGΔBCG1419c immunized mice expressed either a naïve surface phenotype (Fig. 5A) or memory surface phenotype (Fig. 5C), with corresponding declines in spleen and blood. BCG and BCGΔBCG1419c both elicited significant declines in blood effector T_H_ cell (Fig. 5B) and spleen Tfh frequencies (Fig. 5E); however and interestingly, BCG and BCGΔBCG1419c had different effects on the proportion of lung effector and exhausted T_H_ cells: whereas only BCGΔBCG1419c elicited a significant decline in lung effector T_H_ cell frequencies (Fig. 5B), only BCG elicited a significant increase in the lung exhausted T_H_ cell frequencies (Fig. 5D). Similar patterns were observed in the CD8 T cell (T_C_) compartment: in the lung compartment, BCG and BCGΔBCG1419c both elicited an increased frequency of CD8 T cells with a naïve surface phenotype (and corresponding decrease in the spleen), as well as decreases in effector CD8 frequencies in the lung and blood (Fig. 6). Collectively, these data demonstrate that while both BCG and BCGΔBCG1419c elicit increases in the proportion of T cells known to retain memory, BCG-elicited T cells were more likely to have an exhausted phenotype, and BCGΔBCG1419c -elicited T cells were less likely to have an effector phenotype.

### BCG and BCGΔBCG1419c elicited T cell cytokine responses

**Figure 7:**
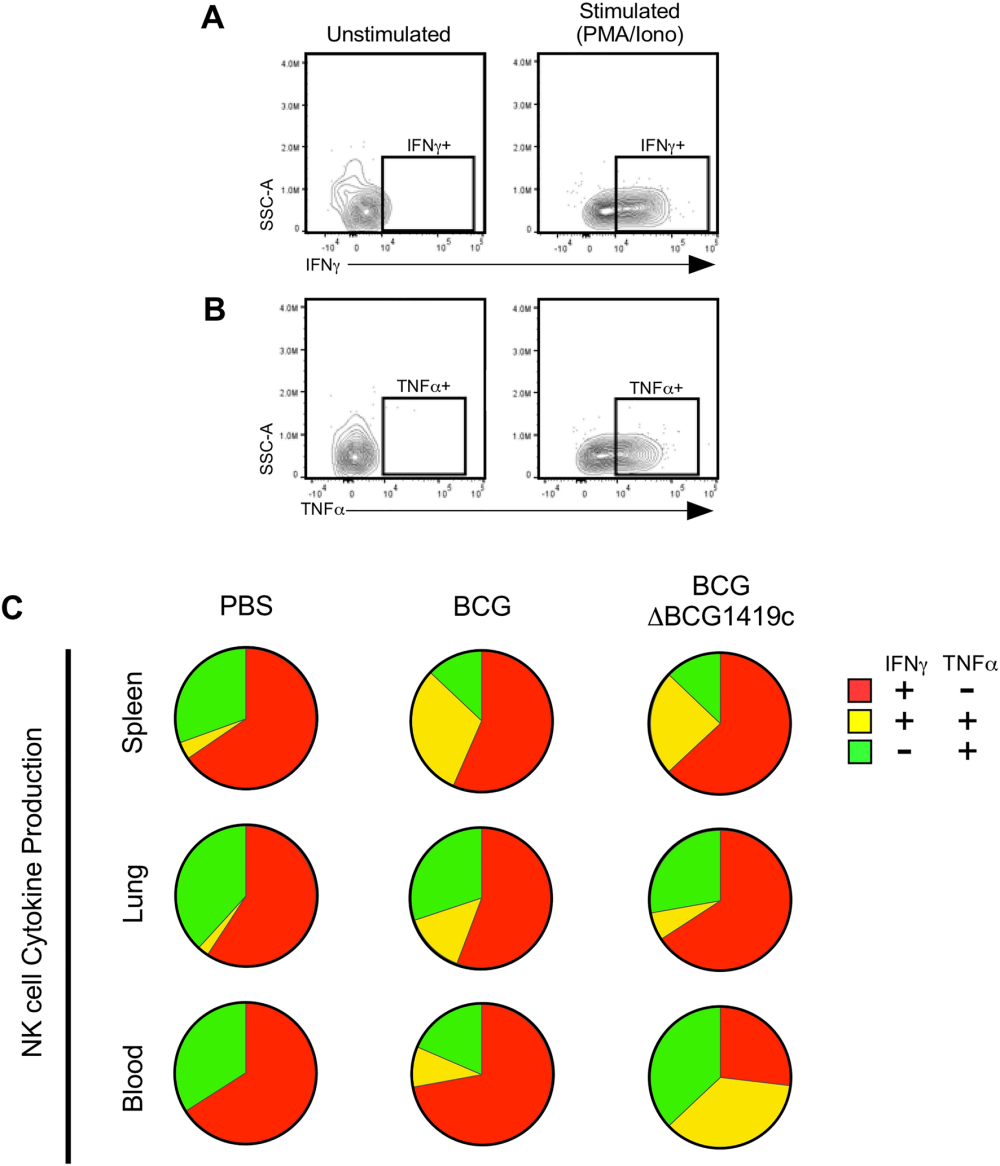
NK cell cytokine production in BCG- and BCGΔBCG1419c-immunized mice. C57BL/6 mice were s.c. immunized with 10^4^ CFU of either BCG or BCGΔBCG1419c, or PBS as a control; 18 days later, mononuclear cells from the spleen, lung and blood were prepared and used for intracellular cytokine staining, following stimulation with PMA/Ionomycin. Shown are (**A**) representative IFNγ staining and (**B**) representative TNFα staining of unstimulated and stimulated mononuclear cells, gated off NK cells; (**C**) Spice analysis of single positive (IFNγ^+^ and TNFα^+^) and double positive (IFNγ^+^TNFα^+^) NK cells in the spleen, lung and blood.

**Figure 8:**
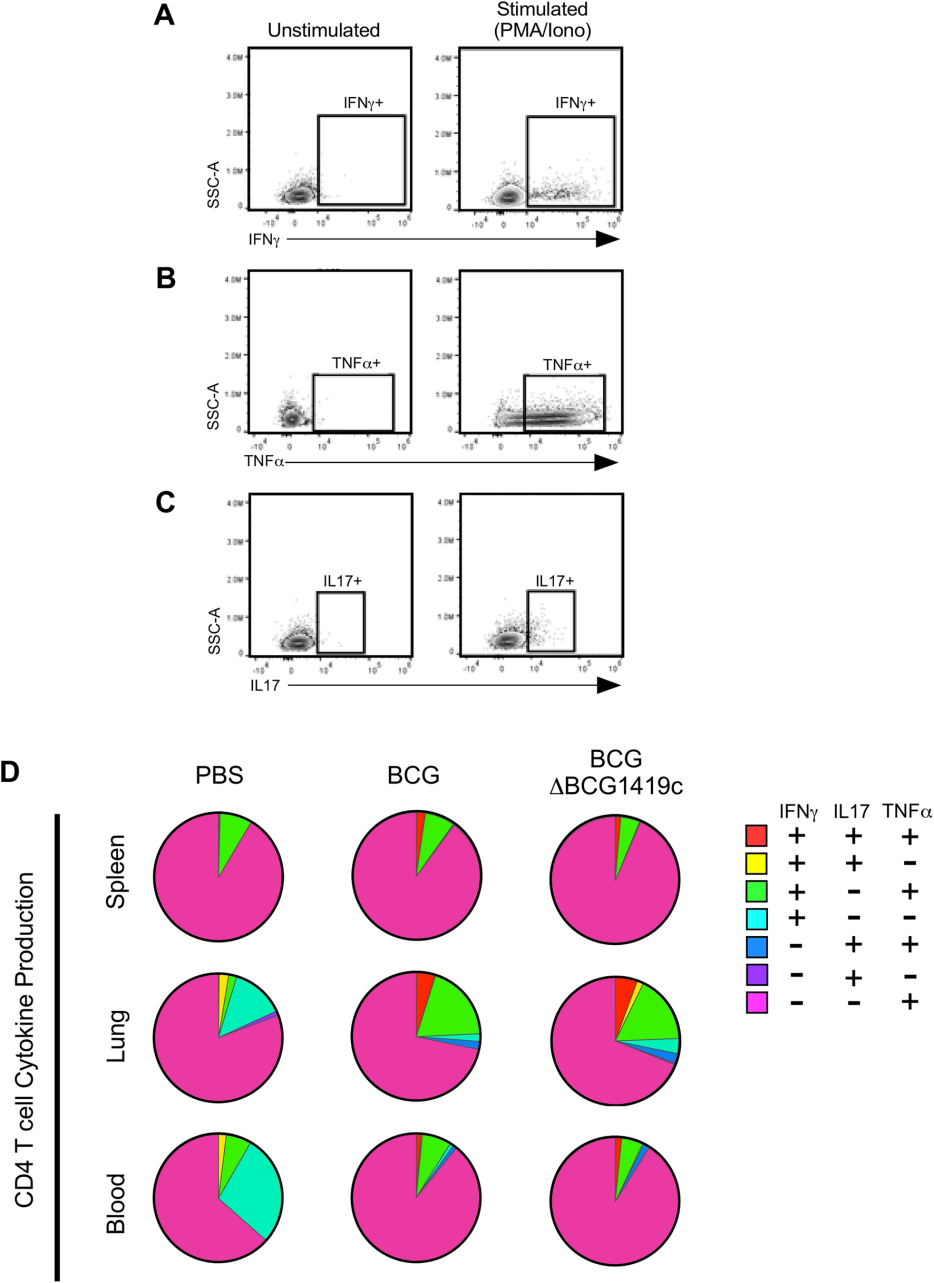
CD4 T cell cytokine production in BCG- and BCGΔBCG1419c-immunized mice. C57BL/6 mice were s.c. immunized with 10^4^ CFU of either BCG or BCGΔBCG1419c, or PBS as a control; 18 days later, mononuclear cells from the spleen, lung and blood were prepared and used for intracellular cytokine staining, following stimulation with PMA/Ionomycin. Shown are (**A**) representative IFNγ staining, (**B**) representative TNFα staining and (**C**) representative IL17 staining of unstimulated and stimulated mononuclear cells, gated off CD4 T cells; (**D**) Spice analysis of single positive (IFNγ^+^, TNFα^+^, IL17^+^ ), double positive (IFNγ^+^IL17^+^, IFNγ^+^TNFα^+^, IL17^+^TNFα^+^), and triple positive (IFNγ^+^, TNFα^+^, IL17^+^) CD4 T cells in the spleen, lung and blood.

**Figure 9:**
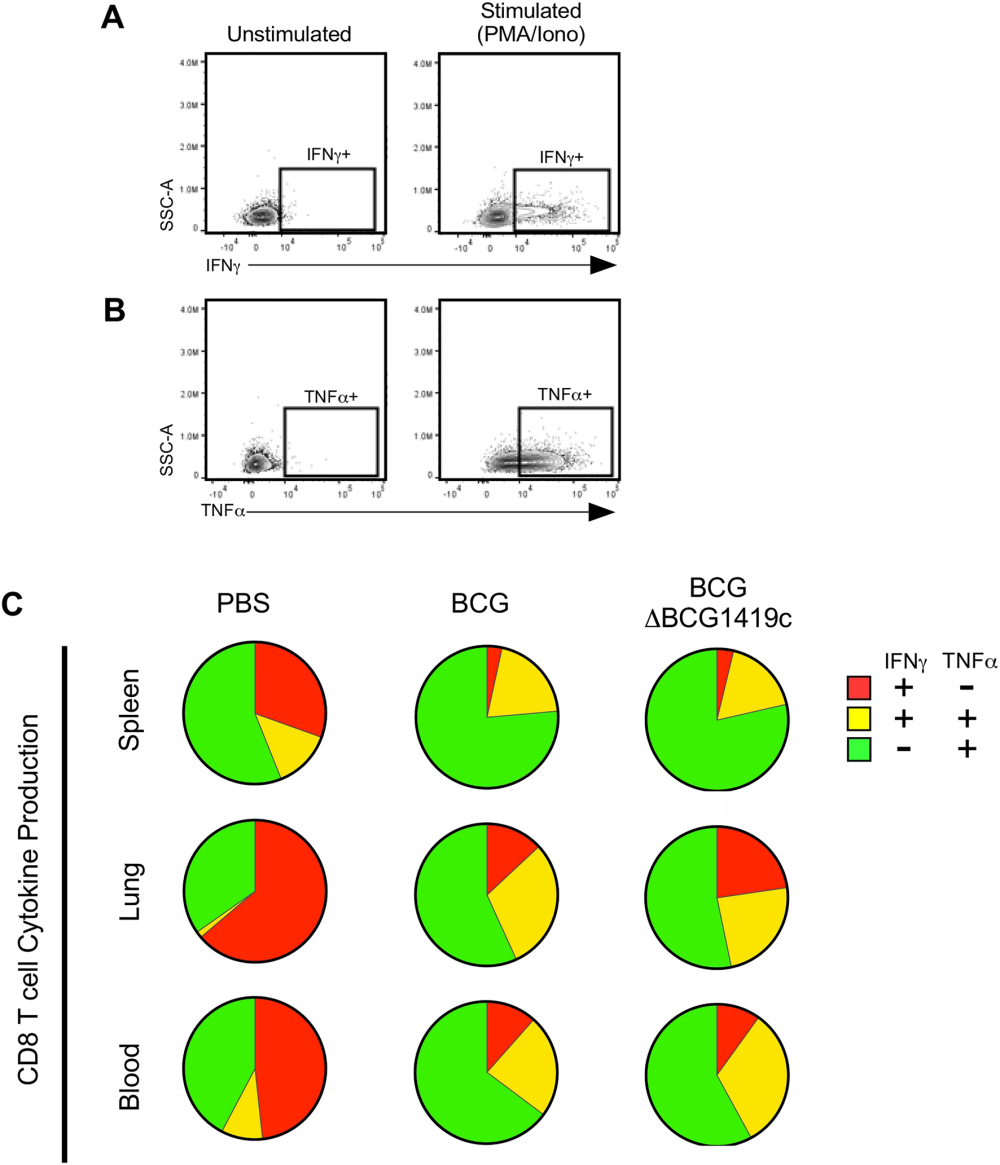
CD8 T cell cytokine production in BCG- and BCGΔBCG1419c-immunized mice. C57BL/6 mice were s.c. immunized with 10^4^ CFU of either BCG or BCGΔBCG1419c, or PBS as a control; 18 days later, mononuclear cells from the spleen, lung and blood were prepared and used for intracellular cytokine staining, following stimulation with PMA/Ionomycin. Shown are (**A**) representative IFNγ staining and (**B**) representative TNFα staining of unstimulated and stimulated mononuclear cells, gated off CD8 T cells; (**C**) Spice analysis of single positive (IFNγ^+^ and TNFα^+^) and double positive (IFNγ^+^TNFα^+^) CD8 T cells in the spleen, lung and blood.

Although there is no universally-accepted cytokine correlate of BCG-elicited protection against *M. tuberculosis* infection^32^, lymphocytes’ capacity for IFNγ, IL17 and TNFα secretion may contribute to BCG vaccine efficacy^33^. To compare the early cytokine profile of BCG- and BCGΔBCG1419c-elicited lymphocytes, the same cell preparations used for surface phenotype analysis (Figs. 2, 3, 4, 5, 6) were stimulated with a polyclonal immunogen (PMA/Ionomycin) and subsequently used for intracellular cytokine staining (ICS) of the NK cell (Fig. 7), CD4 T cell (Fig. 8) and CD8 T cell (Fig. 9) lymphocyte compartments. The results for each compartment are described below.

#### NK cells

NK cell frequencies decline following BCG- or BCGΔBCG1419c-immunization (Fig. 3C); nevertheless, the cytokine profile of those which remain are distinct from those of PBS controls, insomuch there is an expansion of IFNγ^+^TNFα^+^ NK cells in spleen, lung and blood of BCG- and BCGΔBCG1419c-immunized mice (Fig. 7). BCGΔBCG1419c-immunized mice had more single IFNγ^+^cells and BCG-immunized mice had more double IFNγ^+^TNFα^+^ cells in lungs, while BCG induced a higher proportion of single IFNγ^+^cells and BCGΔBCG1419c a more balanced presence of single IFNγ^+^, single TNFα^+^, and double IFNγ^+^TNFα^+^ cells in blood.

#### CD4 T cells

Depicted in Fig. 8A–C are our gating profiles to identify and enumerate T_H_ cells capable of secreting IFNγ (Fig. 8A), IL17 (Fig. 8B) and TNFα (Fig. 8C) in response to polyclonal stimulation. Based upon these flow cytometry data, we were able to distinguish 7 cytokine-producing T_H_ cells: those that were single positive for an individual cytokine (i.e. IFNγ^+^, IL17^+^, or TNFα^+^), double positive for two cytokines (IFNγ^+^IL17^+^, IFNγ^+^TNFα^+^, IL17^+^TNFα^+^), or triple positive for all three cytokines (IFNγ^+^IL17^+^TNFα^+^). The relative proportion of each subset among cytokine producing CD4^+^ T_H_ cells is depicted in Fig. 8D for the spleen, lung and blood of each experimental group. At baseline (i.e. in PBS control mice), cytokine-producing T_H_ cells in all tissues were primarily TNFα^+^, with tissue-specific differences in which subsets were of secondary abundance (spleen: IFNγ^+^TNFα^+^; lung and blood: IFNγ^+^). In the spleen, BCG immunization expanded the representation of IFNγ^+^IL17^+^TNFα^+^ cells; interestingly, the expansion of IFNγ^+^IL17^+^TNFα^+^ was not as large in BCGΔBCG1419c immunized animals and was accompanied by a contraction in IFNγ^+^TNFα^+^ (Fig. 8D top row). A similar contraction of IFNγ^+^TNFα^+^ T_H_ cells was observed in the blood relative to BCG-immunized animals (Fig. 8D bottom row). In the lung, BCG and BCGΔBCG1419c similarly resulted in an expansion of IFNγ^+^IL17^+^TNFα^+^ and IFNγ^+^TNFα^+^, and contraction of IFNγ^+^. IL17^+^TNFα^+^ cells also appeared in BCG and BCGΔBCG1419c lung relative to PBS. Collectively, our analysis of T_H_ cell cytokine profiles suggests that the induction of systemic IFNγ^+^IL17^+^TNFα^+^ and IFNγ^+^TNFα^+^ populations was less robust in BCGΔBCG1419c-immunized animals, relative to BCG-immunized animals, yet in lungs, BCGΔBCG1419c-immunized animals increased the presence of IFNγ^+^IL17^+^ and IFNγ^+^TNFα^+^ populations than BCG; however, none of the above trends were statistically significant.

#### CD8 T cells

Depicted in Fig. 9A,B are our gating profiles to identify and enumerate T_C_ cells capable of secreting IFNγ (Fig. 9A) and TNFα (Fig. 9B) in response to polyclonal stimulation; unlike CD4 T cells, no IL17 expressing CD8 T cells were detected. In PBS controls, cytokine producing CD8 T cells in the spleen were primarily TNFα^+^, and secondarily IFNγ^+^; in response to BCG and BCGΔBCG1419c immunization, the proportion of CD8 T cells that were TNFα^+^ expanded further alongside IFNγ^+^TNFα, at the expense of IFNγ^+^CD8 T cells. Similar expansions of TNFα^+^ and IFNγ^+^ TNFα^+^ cells (and accompanying contractions of IFNγ^+^ CD8 T cells) were observed in the lung and blood of BCG and BCGΔBCG1419c immunized animals. Similar to what we observed for NK cells, BCGΔBCG1419c-immunized mice had more single IFNγ^+^ cells and BCG-immunized mice had more double IFNγ^+^TNFα^+^ cells in lungs; however, as with T_H_ cells none of the above differences between BCG and BCGΔBCG1419c immunized animals were statistically significant.

### The altered early immune response of BCGΔBCG1419c-immunized animals is associated with elevated IL10 production

**Figure 10:**
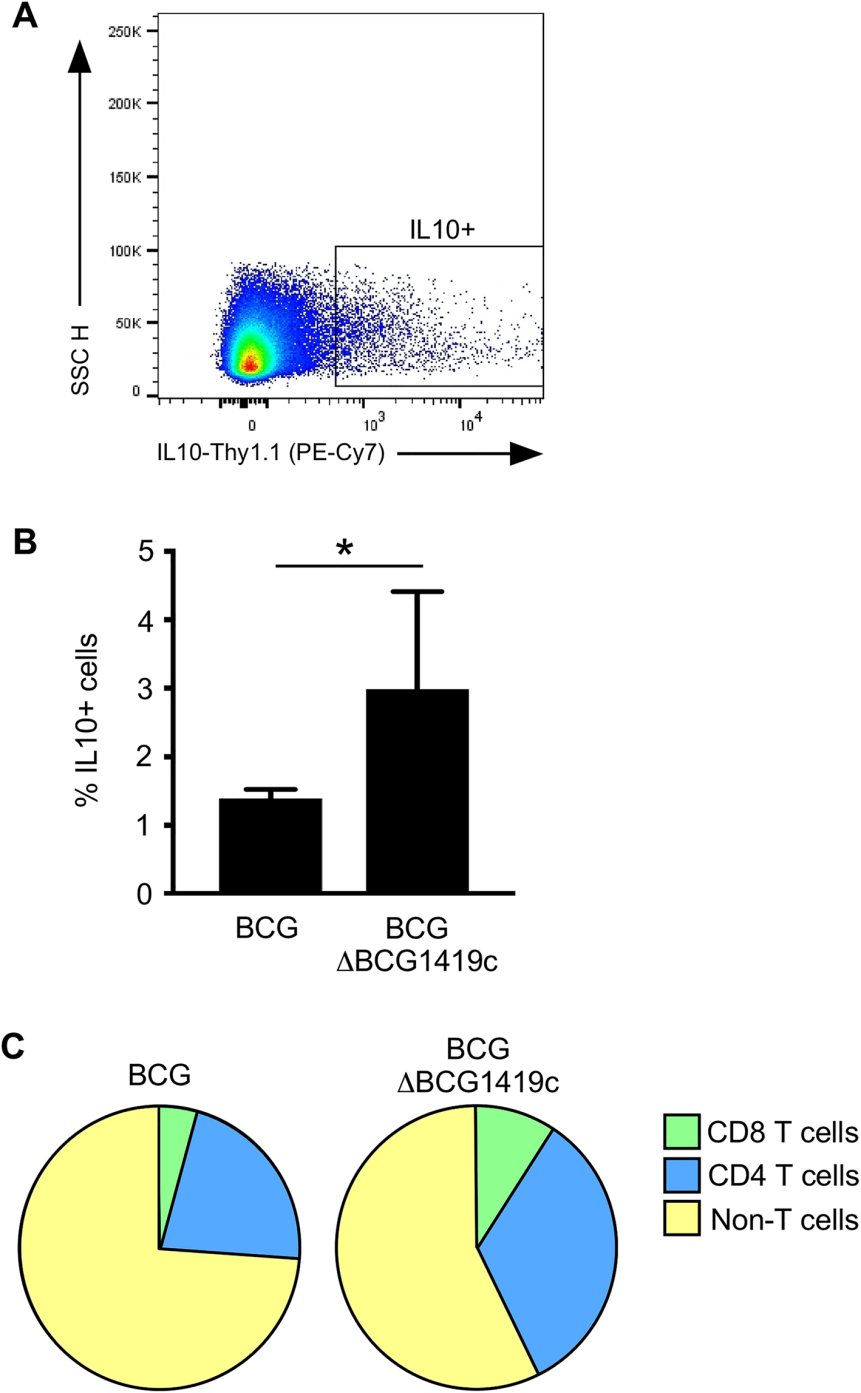
BCGΔBCG1419c-immunization elicits a higher frequency of IL10-producing T cells. IL10 reporter mice were s.c. immunized with 10^4^ CFU of BCG or BCGΔBCG1419c; 14 days later, splenocytes from each group were stained for Thy1.1 (the transgene reporter of IL10 production) and T cell markers CD4 and CD8. Shown are (**A**) representative side scatter (SSC) and Thy1.1 staining from BCG-immunized mice, (**B**) the frequency of IL10^+^ splenocytes in BCG- and BCGΔBCG1419c-immunized mice (mean + SD; 3 mice per group), and (**C**) the relative proportion of IL10^+^ cells that are CD4 T cells, CD8 T cells or non-T cells. Asterisks represent a statistically significant difference between indicated comparison (*p ≤ 0.05).

IL10 suppresses T_H_1/T_H_17 differentiation following BCG immunization^34^. At post-immunization day 18, BCGΔBCG1419c-tended trended (albeit insignificantly) toward eliciting fewer IFNγ- and IL17-producing T_H_ cells (Fig. 8D), as well as a greater shift away from the more pro-inflammatory “effector” T_H_ phenotype (Fig. 5B); therefore, we predicted that BCGΔBCG1419c-immunized mice would—relative to BCG-immunized mice—have higher frequencies of systemic IL10-producing cells prior to day 18. To test this prediction, we immunized transgenic VertX mice that express surface Thy1.1 concomitant to IL10 expression^26^, permitting the identification of IL10 producing cells by flow cytometry staining with anti-Thy1.1 (Fig. 10A). VertX mice were immunized with BCG or BCGΔBCG1419c in a manner identical to that used for C57BL/6 mice; the spleen was removed at 14 days post-immunization. The results of this study are shown in Fig. 10 and demonstrate that BCGΔBCG1419c elicits higher frequencies of IL10^+^ cells in the spleen (Fig. 10B), and that a larger proportion of these IL10^+^ cells were CD4^+^ T cells and CD8^+^ T cells (Fig. 10C). Overall, these data demonstrate that BCGΔBCG1419c elicits higher frequencies of IL10 producing T cells, which may account for less inflammatory response of BCGΔBCG1419c-immunized animals relative to wild type BCG-immunized animals.

## Discussion

BCG was developed as a TB vaccine in the early 1900s by Calmette and Guerin, following serial passage of *M. bovis* for eleven years (~ 230 passages) on bile-imbibed potato slices^35^. Relative to *M. bovis*, the resulting strain (i.e. BCG) was avirulent in multiple animal species and unable to cause tuberculosis. We now know this attenuation stems in part from the loss of three genomic regions of difference (RD1, RD2 and RD3) which encode a number of virulence factors (e.g. ESX)^36^. Despite several starts and stops due to safety concerns, human BCG trials moved forward in a number of countries (UK, US, India) with differing conclusions regarding its protective efficacy, which ranges from 0 to 80% depending on the study^37^. Still a matter of debate, the reasons for BCG’s variable efficacy in different countries and populations include different preparation methods, genetic drift over time, and environmental mycobacteria which themselves confer some protection against TB and thus mask any effect of BCG^38^. Regardless of the reason(s), there is near-universal agreement that despite BCG’s success a TB vaccine with better efficacy is needed to meet WHO benchmarks for reducing TB prevalence^5^. Partly due to lack of full efficacy of current BCG, an estimated 2 billion people are thought to harbor *M. tuberculosis* latent infection (LTBI). It has been estimated that by 2050, up to 75% percent of new TB cases will be due to reactivation from LTBI, rather than new infections, in China, one of the countries with the highest number of TB cases (including MDR-TB)^39^. In fact, a model that includes data from China, South Africa, and India, suggested that vaccines preventing disease in *M. tuberculosis*-infected populations would have the greatest impact by 2050 (10-year, 70% efficacy against disease, incidence rate reduction 51%, 52%, and 54% in China, South Africa, and India, respectively)^40^.

Like many mycobacterial species, BCG is capable of forming biofilms which impact upon in vitro behavior and in vivo persistence. Biofilms comprise a community of bacteria surrounded by an extracellular matrix produced by the constituent species, are generally more resistant to environmental stress, and enable persistence in environmental reservoirs and biomaterials^41^. In vitro, mycobacteria biofilm formation manifests as pellicles at the media-air interface^42,43^, which in BCG follow a defined genetic program^44^. Pellicles have an altered transcriptional profile relative to planktonic bacteria and are enriched in antibiotic drug resistant cells or persisters^45–47^. The in vivo contribution of mycobacteria biofilm formation to lung disease is often a topic of debate since their presence is not obvious upon histo-pathological examination of infected lungs (e.g. by acid fast staining), and mycobacterial pathogenesis involves intracellular survival within alveolar phagocytes (at least during early phases of disease) rather than extracellular survival on airway surfaces, which is more typical of *Pseudomonas aeruginosa* and other canonical biofilm-forming pathogens. That said, mycobacteria which are deficient in biofilm formation—due to either genetic manipulation or natural variation—generally exhibit attenuated virulence and elicit different inflammatory responses during in vivo infection models^42,48^. Our understanding of the relationship between biofilm formation and mycobacterial pathogenesis is therefore far from complete.

The BCGΔBCG1419c strain was generated in part to test the relationship between BCG’s ability to form biofilms and its vaccine efficacy^20^. The *BCG1419c* gene encodes a cyclic di-GMP phosphodiesterase (PDE) that normally functions to hydrolyze Bis-(3′-5′)-cyclic dimeric GMP (c-di-GMP), a second messenger which promotes the biofilm phase of bacterial growth^49^. In the absence of *BCG1419c*, the deletion mutant BCGΔBCG1419c exhibits an altered colony morphology, higher biofilm production and extended survival in the lungs and spleen of immunocompetent mice, relative to parental BCG or complemented mutants^20^ when used for intravenous infection of BALB/c mice. Relative to BCG immunized controls and at 6 months post-Mtb challenge, those immunized with BCGΔBCG1419c also exhibit significantly reduced lung histopathological scores, as well as reduced lung IL6 and TNFα protein levels^15^. Since IL-6 and TNF-α may contribute to lung pathology in chronic disease settings, these data implicate BCG1419c-dependent hydrolysis of c-di-GMP in conditioning the long-term TB response of vaccinated animals. Importantly, the bacterial second messenger c-di-GMP is a ligand for the stimulator of interferon (IFN) genes (STING) signaling pathway via the tank-binding kinase-1 (TBK1)-interferon regulatory factor 3 (IRF3) cascade, which results in production of type I IFN- and NF-κB-mediated cytokines^50,51^. These STING agonists have shown potential use as novel vaccine adjuvants, as evidenced by their immunostimulatory properties, due to their ability to increase antigen-specific T cell and humoral responses^52,53^. Because of the deletion of the c-di-GMP phosphodiesterase-encoding gene *BCG1419c*^54,55^ we expect the BCGΔBCG1419c vaccine candidate to produce more c-di-GMP, therefore modulating the type I IFN response to possibly increase its efficacy against TB. Further to this, BCGΔBCG1419c has a modified proteomic repertoire compared with parental BCG, including changes in cellular and secreted antigenic proteins^54^ which could also lead to a differential immune response which ultimately impact, in a positive manner, protection against pulmonary TB.

Given that BCGΔBCG1419c immunized mice have less lung pathology following TB challenge, suggesting an improvement to prevent disease, we undertook the above study to determine if there are early immunological events which coincide with its long term effects. Our results demonstrate that while there are many similarities in the early innate and adaptive response to BCG and BCGΔBCG1419c, there are also notable differences in the innate and adaptive compartments. Among innate lineages, whereas both BCG and BCGΔBCG1419c elicited strong reductions in NK cell frequency across all tissues examined (Fig. 3C), only BCGΔBCG1419c elicited significant increases in circulating monocytes (Fig. 3D) and reductions in lung Gr1 cells (Fig. 3E). Among adaptive lineages, both BCG and BCGΔBCG1419c elicited significant increases in lung CD4^+^ T cell frequencies (Fig. 4A), those induced by BCG were more likely to exhibit an exhausted phenotype relative to those induced by BCGΔBCG1419c (Fig. 5D). BCGΔBCG1419c elicited CD4 T cells were also less likely to exhibit a pro-inflammatory effector phenotype (Fig. 5B). Importantly, the CD4 and CD8 T cell response elicited by BCGΔBCG1419c were also more likely to contain IL10-producing cells (Fig. 10C), which may underlie the less inflammatory nature of BCGΔBCG1419c during chronic TB stages since IL10 suppresses via antigen presentation, via retention of mycobacterial peptide:MHC-II complexes in endosomal fractions (away from the cell surface)^56^ and/or negative regulation of costimulatory molecule expression^57,58^. We have shown that BCGΔBCG1419c produced less DnaK, HbhA, PstS2, 35KDa antigen, and AcpM (a protein involved in synthesis of mycolic acids) in biofilm cultures compared with parental BCG^14^, possibly contributing to its less inflammatory nature. Still to be determined is whether the immune response elicited by BCGΔBCG1419c differs from that elicited by BCG due to either prolonged antigen presentation or altered lipid profiles, which contribute to mycobacteria immunogenicity^59^.

As with any bacteria-driven inflammatory response, the BCG-immunized host must balance the generation of robust immunity with the potential for detrimental, non-specific effects of excess cytokine production. Here, we used the mouse model to demonstrate that the early immunological events associated with BCGΔBCG1419c immunization are less inflammatory relative to BCG. This is important because BCGΔBCG1419c’s efficacy in controlling *M. tuberculosis* replication in lungs is comparable or superior to that of BCG, and more protective against TB-associated immunopathology in mice. Given the reactogenicity of BCG in immunized individuals, which commonly manifests as fever, headache and swollen lymph nodes, BCGΔBCG1419c’s unique immunological profile may prove important in TB endemic regions where parents of young children are increasingly hesitant of inflammatory vaccine administration, regardless of the vaccine efficacy^9–12^.
